# The complete chloroplast genome of a rare species in Korea, *Lilium Dauricum* Ker Gawl

**DOI:** 10.1080/23802359.2019.1676174

**Published:** 2019-10-15

**Authors:** Rahul Vasudeo Ramekar, Kyong-Cheul Park, Myounghai Kwak, Ik-Young Choi

**Affiliations:** a Department of Agriculture and Life Industry, Kangwon National University, Chuncheon, South Korea;; b Plant Resources Division, National Institute of Biological Resources, Incheon, South Korea

**Keywords:** Chloroplast genome, *Lilium dauricum*, phylogenetic analysis

## Abstract

*Lilium dauricum* Ker Gawler is a wild lily species that belongs to section Sinomartagon and is one of the ancestors of the Asiatic hybrid lilies. Unique traits such as disease resistance and early flowering make *L. dauricum* a desirable resource for interspecific hybridization. However, in Korea, the natural resources of *L. dauricum* are being exhausted by excessive exploitation and require urgent conservation. In this study, the complete chloroplast genome of *L. dauricum* was generated using Illumina paired-end sequencing technology, and its structure was compared with that of other *Lilium* species. The chloroplast genome was 152,063 bp long, with a typical quadripartite structure including a pair of inverted repeat regions (26,492 bp) separated by a large (81,485 bp) and small (17,584 bp) single-copy (SC) region. The genome encodes 131 different genes, including 85 protein-coding genes, 38 tRNA genes, and 8 rRNA genes. A phylogenetic analysis strongly supported the relationship of *L. dauricum* with other members of Sinomartagon and Martagon lilies.

## Introduction

*Lilium dauricum* Ker Gawler (genus *Lilium*) is a perennial bulb-producing flowering plant known for its high degree of resistance to *Fusarium oxysporum* var *lilii*, and exhibits early flowering, making it a valuable genitor for both interspecific and intersectional hybridizationhybridisation (Löffler et al. 1996). In Korea, the considerable economic value of *L. dauricum* has led to its overexploitation, causing a severe reduction in the size of its natural population.

Here, we report on the complete chloroplast (cp) genome sequence of *L. dauricum*. Plant material collected from Odesan Mountain, Gangwon-do, South Korea (37.46.42N, 128.36.02E) under accession number 2015-10 high-throughput sequencing was performed using an Illumina HiSeq 4000 platform. The raw reads were quality trimmed by Trimmomatic (Bolger et al. 2014), and high-quality reads were assembled by the Newbler assembler. The representative cp contigs were extracted, ordered, and merged into a single draft sequence by comparing with the already available cp genomes of *Lilium* species. The structural features and genes were predicted using the DOGMA program (Wyman et al. 2004), manual curation based on BLAST searches and ARTEMIS software (Rutherford et al. 2000). We have submitted the assembled and annotated sequence to GenBank under the accession number MK879804.

**Figure 1.**
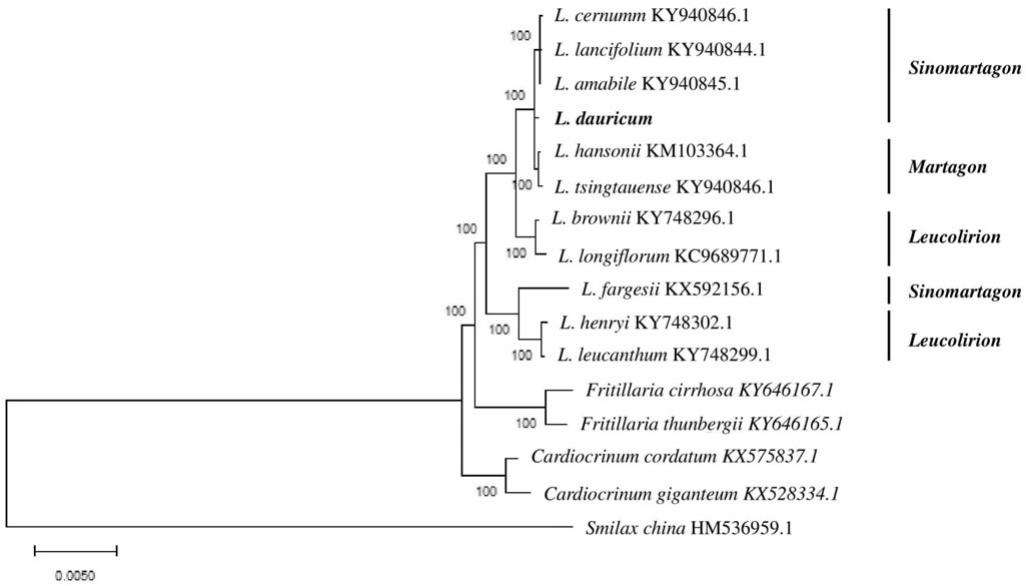
Molecular phylogenetic tree of the Liliaceae family based on the complete cp genome of 16 species.

To ascertain the phylogenetic status of *L. dauricum*, the complete cp genome of 15 species in the family Liliaceae, including 11 representative species from the genus *Lilium* and two species each from *Fritillaria* and *Cardiocrinum*, and that of *Smilax china* as an outgroup were selected. A neighbour-joining (NJ) tree was constructed with Mega 6.0 using 1000 bootstrap replicates (Tamura et al. 2013) clustered the *Lilium* species into two groups. One group comprised sections *Sinomartagon* (*L. cernuum, L. lancifolium, L. amabile,* and *L. dauricum*), *Martagon* (*L. hansonii* and *L. tsingtauense*) and *Leucolirion* (*L. brownii* and *L. longiflorum*). Another group comprised *Sinomartagon* (*L. fargesii*) and *Leucolirion* (*L. henryi* and *L. leucanthum*) (Figure 1). *Fritillaria* and *Cardiocrinum* were placed in a distinct cluster, and *Lilium* and *Fritillaria* were strongly supported as sister taxa. *L. dauricum* together with sections *Sinomartagon* (*L. cernuum, L. lancifolium, L. amabile,* and *L. dauricum*) and *Martagon* (*L. hansonii* and *L. tsingtauense*) formed a monophyletic clade with a high bootstrap value, indicating a close relationship among these species.

## References

[CIT0001] Bolger AM, Lohse M, Usadel B 2014 Trimmomatic: a flexible trimmer for Illumina sequence data. Bioinformatics. 30(15):2114–2120. 24695404 10.1093/bioinformatics/btu170 PMC4103590

[CIT0005] Löffler HJM, Meijer H, Straathof TP, van Tuyl JM 1996 Segregation of Fusarium resistance in an interspecific cross between *Lilium longiflorum* and *Lilium dauricum*. Acta Hortic. 414:203–208.

[CIT0006] Rutherford K, Parkhill J, Crook J, Horsnell T, Rice P, Rajandream MA, Barrell B 2000 Artemis: sequence visualization and annotation. Bioinformatics. 16(10):944–945. 11120685 10.1093/bioinformatics/16.10.944

[CIT0007] Tamura K, Stecher G, Peterson D, Filipski A, Kumar S 2013 MEGA6: molecular evolutionary genetics analysis version 6.0. Mol Biol Evol. 30(12):2725–2729. 24132122 10.1093/molbev/mst197 PMC3840312

[CIT0008] Wyman SK, Jansen RK, Boore JL 2004 Automatic annotation of organellar genomes with DOGMA. Bioinformatics. 20(17):3252–3255. 15180927 10.1093/bioinformatics/bth352

